# The modular small-angle X-ray scattering data correction sequence

**DOI:** 10.1107/S1600576717015096

**Published:** 2017-11-29

**Authors:** B. R. Pauw, A. J. Smith, T. Snow, N. J. Terrill, A. F. Thünemann

**Affiliations:** a Bundesanstalt für Materialforschung und -prüfung (BAM), 12205 Berlin, Germany; b Diamond Light Source Ltd, Diamond House, Harwell Science and Innovation Campus, Didcot, Oxfordshire OX11 0DE, UK

**Keywords:** small-angle scattering, accuracy, methodology, data correction

## Abstract

Data correction is probably the least favourite activity amongst users experimenting with small-angle X-ray scattering: if it is not done sufficiently well, this may become evident only during the data analysis stage, necessitating the repetition of the data corrections from scratch. A recommended comprehensive sequence of elementary data correction steps is presented here to alleviate the difficulties associated with data correction, both in the laboratory and at the synchrotron. When applied in the proposed order to the raw signals, the resulting absolute scattering cross section will provide a high degree of accuracy for a very wide range of samples, with its values accompanied by uncertainty estimates. The method can be applied without modification to any pinhole-collimated instruments with photon-counting direct-detection area detectors.

## Abstract 2

A data correction sequence is presented, consisting of ordered elementary steps that extract the small-angle X-ray scattering cross section from the original detector signal(s). It is applicable to a wide range of samples, including solids and dispersions.

## 1. Introduction   

Attaining a high standard for data quality is paramount for any insightful analysis. This is particularly important for small-angle scattering, where the largely featureless scattering patterns may easily be insufficiently corrected and/or over- or under-fitted by an inexperienced user. Previous work on data correction procedures tended to follow an *ad* *hoc* approach, incorporating a limited incomplete subset of the available corrections in an integrated correction step. Such methods offer neither flexibility nor the ability to trace the effects of a given correction step (Stothart, 1987 ▸; Strunz *et al.*, 2000 ▸; Dreiss *et al.*, 2006 ▸).

This can be resolved with the provision of a comprehensive modular set of elementary two-dimensional data correction steps. These steps can be chained together to form a bespoke and complete correction sequence for a given instrument or sample in any small-angle scattering laboratory or synchrotron. When enhanced with the ability to estimate and propagate well founded uncertainty estimates^1^ on (at least) the resulting scattering cross-section values, it allows said laboratory to rapidly evaluate and select the subset of significant corrections for their experiments or instruments. The two-dimensional nature of the corrections renders them appropriate for isotropic as well as anisotropically scattering samples, increasing their universality.

While the majority of these individual data correction steps have already been comprehensively collated for experiments using both X-rays (Pauw, 2013 ▸; Feigin *et al.*, 1987 ▸) and neutrons (Hollamby, 2013 ▸; Brûlet *et al.*, 2007 ▸), the recommended order in which they are to be applied has so far not been published. For X-ray experiments in particular, this was due to a historical lack of software that might benefit from such a scheme, and because such a sequence had not been finalized until now. With the recent emergence of various two-dimensional data correction software packages capable of performing such comprehensive modular corrections (Arnold *et al.*, 2014 ▸; Basham *et al.*, 2015 ▸; Benecke *et al.*, 2014 ▸; Filik *et al.*, 2017 ▸; Girardot *et al.*, 2017 ▸; Nielsen *et al.*, 2009 ▸; Taché *et al.*, 2017 ▸; Solé *et al.*, 2017 ▸), establishing a recommended starting point for implementing such a data correction schema seems pertinent.

This work, therefore, provides a near universally applicable, ordered schema of corrections with which the absolute scattering cross section can be extracted from raw detector information in a consistent and reproducible manner. This scattering cross section is determined multidimensionally (typically two dimensionally) and can optionally be reduced to one dimension, *i.e.* where the averaged scattering cross section is determined as a function of scattering vector **Q** or azimuthal angle χ. This result can then be fed to a wide range of data analysis programs for morphological elucidation, such as *ATSAS*, *GIFT*, *Irena*, *McSAS*, *SASfit* and *SASView*, to name but a few (Franke *et al.*, 2017 ▸; Glatter, 1980 ▸; Ilavsky & Jemian, 2009 ▸; Bressler *et al.*, 2015 ▸; Breßler *et al.*, 2015 ▸; Alina *et al.*, 2017 ▸). This schema can be used as the core of a data correction software package or as a reference correction sequence against which (faster) alternatives can be proven. It is hoped that adherence to this schema will improve the already exemplary comparability of results obtained at different instruments (Pauw *et al.*, 2017 ▸; Rennie *et al.*, 2013 ▸).

The near universality of the scheme implies that it is applicable to most sample types (*cf.* Table 1 ▸). This property stems from its three-stage correction process: The first two stages of the correction process are sufficient to correct homogeneous samples (single phase), and the addition of the third stage means it can also extract a particular scattering signal from both dilute and dense heterogeneous samples. Examples of such investigations include size and shape investigations of colloidal dispersions (Wagner *et al.*, 2000 ▸), the physical state of self-assembled systems (Soni *et al.*, 2006 ▸), the elucidation of structural and orientational details from polymers (Heeley *et al.*, 2005 ▸), and protein sizing in solution (Rambo & Tainer, 2013 ▸). Additionally it is possible to use this schema to extract the scattering of dynamic systems such as evaporative drying (Gu *et al.*, 2016 ▸), *in situ* chemical reactions (Chen *et al.*, 2015 ▸), phase or state changes experienced by soft-matter systems with respect to change from external influences and changes (Bulpett *et al.*, 2015 ▸), and deterioration in systems exposed to stress, strain, wear or age (Turunen *et al.*, 2016 ▸).

Over the past few years, most elements of the schema have been developed, tested and refined in practice, on both laboratory- and synchrotron-based small-angle X-ray scattering (SAXS) instruments. Its development has focused on modern instruments, and a direct-detection photon-counting detector is, therefore, highly recommended in order to achieve the best results. The use of such a photon-counting detector in the following is implicit: the data correction steps necessary to compensate for the inadequacies of other detector types have been omitted for brevity.

We here present the ordered schema, each correction’s abbreviation is briefly described, and the reasoning behind their chosen position in the sequence is clarified.

## 2. The schema   

**Figure 1:**
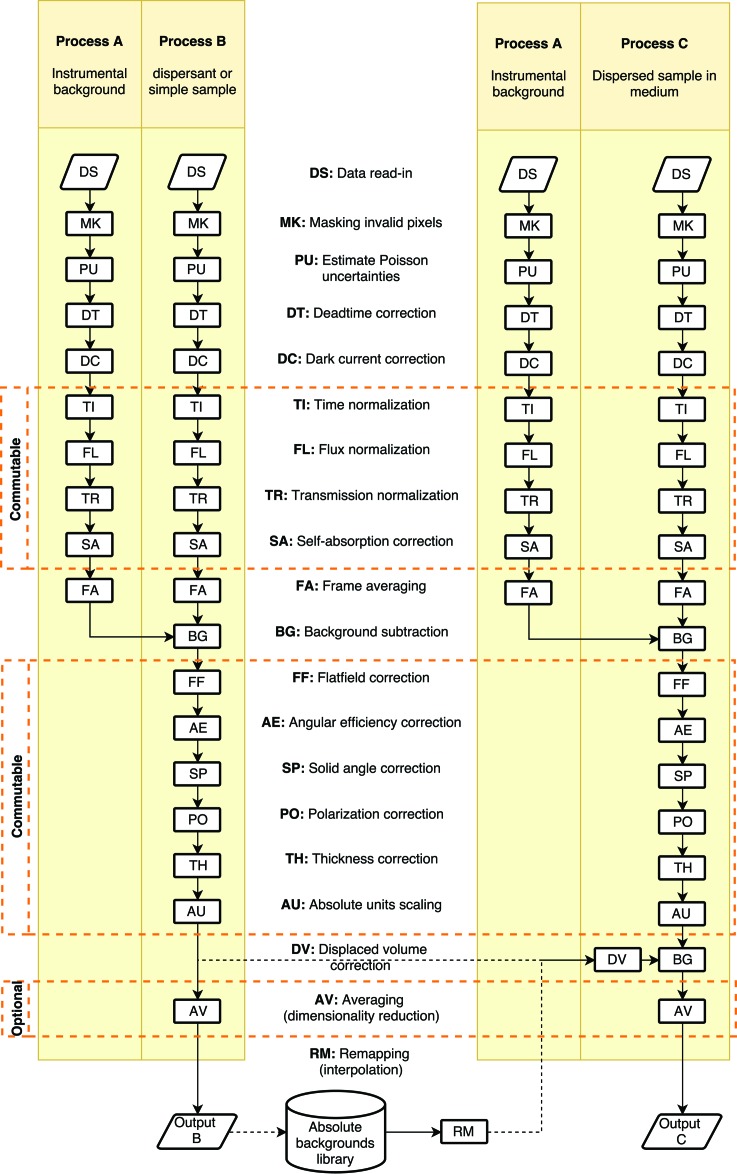
The recommended data correction sequence for most types of samples. Output B for solids is the corrected data in absolute units, and for dispersions it is the dispersant (solvent) scattering in absolute units. Output C for dispersions is the sample scattering in absolute units. The azimuthal averaging step can be considered for isotropically scattering samples.

The recommended data correction schema covering a wide range of practical samples is presented in Fig. 1 ▸. This data correction schema consists of three different series of corrections, denoted as ‘Process A’, ‘Process B’ and ‘Process C’. The measurements to be used in a particular process depend on the object of interest, with many examples given in Table 1 ▸. In general, Process A should be used on a measurement of the instrumental background (including empty sample cells when sample cells are used). Process B should be applied to (1) measurement of a non-dispersed material of interest or (2) a dispersant, be it either solid, liquid or gas. In the second case, Process C is used for the measurement of the dispersant with the analyte. The output from Process C is then the absolute scattering power of the analyte alone, and the output from Process B is the absolute scattering power of the dispersant.

When applied to homogeneous polymer films, for example, the instrumental background measurement (measured with nothing at the sample position) is inserted in Process A and the polymer film measurement in Process B. From the output of that process, ‘Output B’, we then obtain the polymer film scattering cross section in absolute units. If, however, determination of the scattering arising from nanoparticles embedded within that polymer film were desired, then it would be necessary to collect a measurement of the non-nanoparticle-containing film for Process B and the nanoparticle-containing film in Process C. Output C will then be the scattering power of the embedded nanoparticles alone.

Likewise, for a dispersion of nanoparticles in a solvent contained in a capillary, measurement of the empty capillary would be used in Process A, the solvent in capillary in Process B and the dispersion in capillary in Process C. This leads to the acquisition of the absolute scattering cross section for the solvent in Output B and that of the pure analyte in Output C. The advantage of this approach is that the solvent scattering may be reused for other samples measured using the same energy by adding this to a solvent scattering library. A disadvantage of this approach is that the uncertainties of several operations are added twice to the scattering cross section for dispersions (Output C), such as the uncertainties on the flatfield and polarization corrections, which will be discussed below.

Note that the same capillary should be used for both Process A and B, when obtaining the dispersant scattering cross section. Likewise, the capillary used for the set of Processes A and C, *i.e.* when collecting the measurement of the dispersant with analyte, should also be identical. These conditions, therefore, necessitate the use of reusable containers or flow-through cells.

## 3. The steps and reasoning behind the sequence   

The mathematical expressions for most of the corrections below are described by Pauw (2013 ▸), with the remainder given in the appendices. Here, we focus on the justification of the steps and highlight the position dependency of some of them.

(i) **DS** (data read-in): before starting any data corrections, the data must be read in correctly, where necessary compensating for the data storage peculiarities (Knudsen *et al.*, 2013 ▸).

(ii) **MK** (masking): invalid pixels are masked so they are not considered in the following corrections.

(iii) **PU** (Poisson uncertainty estimator): the Poisson (counting) uncertainty needs to be calculated on the number of detected photons, and therefore this step is carried out before the deadtime, dark current or flatfield corrections. Some detectors automatically apply corrections and will require (software) adjustments before the uncorrected counts can be retrieved.^2^

(iv) **DT** (deadtime): the signal is subsequently corrected for the detector deadtime, returning the estimated number of photons arriving at each pixel on the basis of the detected count rate.

(v) **DC** (dark current): the subtraction of natural background radiation (including the steady flow of cosmic rays) forms the dominant component of the dark current correction. With the aforementioned recommended detector type, we should not see any significant contribution of the time-independent and flux-dependent dark current components.

(vi) **TI** (time): a normalization to render the measurement independent of the measurement duration.

(vii) **FL** (flux): a normalization to make the measurement independent of the incident beam flux.

(viii) **TR** (transmission): a scaling correction, correcting for the probability of absorption (and only absorption) within the sample. The transmission should, ideally, be calculated by dividing the flux of all transmitted, scattered and diffracted radiation by the incident flux. Note that the quality of the obtained scattering cross section is very strongly dependent on the quality of the transmission factor (in particular when the background subtraction operation is applied), and an accuracy of >99% should be aimed for.

(ix) **SA** (self-absorption): the sample self-absorption is the correction for the increased probability of scattered rays that are absorbed as they travel through slightly increased amounts of sample after the scattering event. This correction needs to be performed after the transmission correction: it represents a direction-dependent modification to the transmission correction and does not replace the TR correction itself. It is feasible to implement and use for samples of plate-like geometry (only), with the plate surface perpendicular to the X-ray beam direction. It is related and therefore placed next to the transmission correction.

(x) **FA** (frame averaging): it is recommended for photon-counting direct-detection systems that the measurements be split up into multiple shorter frames. This avoids saturation of the per-pixel counters and allows for temporal variations in signal due to beam, instrument or sample instabilities to be recognized. When no significant variation between the frames is observed, they can be averaged in this step. When the averaging is, furthermore, weighted by the scattering signal uncertainty of each pixel in each frame, frames collected with different exposure times can be averaged to obtain a high dynamic range (HDR) scattering signal. Any saturated pixels for a long-exposure frame will then have been masked by the MK process.

(xi) **BG** (background subtraction): the subtraction of the background signal is done only after the measurement-dependent corrections have taken place, as the various parameters (transmission, flux, time and therefore dark current in particular) may differ.

(xii) **FF** (flatfield): the flatfield correction, a multiplication matrix normalized to 1, corrects for inter-pixel sensitivity differences. An example of its magnitude in modern detectors is given by Wernecke *et al.* (2014 ▸).

(xiii) **AE** (angular efficiency): this correction compensates for variations in the detector efficiency depending on the photon angle of incidence onto the detector surface. It is detailed in Appendix *C* [App appc]. This is the last of the corrections for detector imperfections.

(xiv) **SP** (solid angle): a (geometric) correction for the solid angle subtended by each pixel. This can be calculated on the basis of the instrument geometry alone.

(xv) **PO** (polarization): the polarization correction com­pensates for differences in the probability of scattering events, both for polarized and for unpolarized beams. In the latter case, it is an azimuthally uniform (isotropic) correction. The polarization correction is performed before the second background subtraction, so that older dispersant measurements can still be used for correction of future samples.

(xvi) **TH** (thickness): the thickness correction normalizes the data to units of reciprocal length. Note that the thickness used in this correction is the thickness of the solid sample or the liquid phase for dispersions only. A derivation supporting this is provided in Appendix *D* [App appd].

(xvii) **AU** (absolute units): the absolute units correction scales the data to units of scattering cross section, the fraction of radiation that is scattered per length of material per solid angle. This is commonly reported in units of 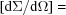 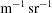 or 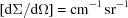.

(xviii) **DV** (displaced volume): this correction has not been included by Pauw (2013 ▸), but is described in Appendix *B* [App appb]. This correction can be applied for dispersions with high volume fractions of analyte, but must be done on the solvent scattering signal only.

(xix) **RM** (remapping): for background subtraction operations using a previously stored dataset, a mapping or interpolation operation may be necessary to match the dimensionality and angular range of the dataset in the processing step.

(xx) **AV** (averaging): this optional step reduces the dimensionality and size of the dataset, typically from two dimensions to a limited number of data points in one dimension. This can be done azimuthally (to obtain 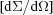 *versus* *Q*) or radially (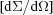 *versus* χ). The azimuthal averaging is suitable for isotropic data, whereas the radial averaging is typically applied to anisotropic data over a limited radial range, to extract a degree of orientation. This is commonly performed in fibre diffraction experiments.

Note that the averaging from two dimensions to one dimension is performed after the background subtraction step as (1) it is optional and (2) the background subtraction process in particular can subtract anisotropic signals such as flares. In that case, the practical uncertainty estimated during the averaging procedure is reduced if the background subtraction is done in two dimensions rather than after averaging, as shown in Appendix *A* [App appa]. Note also that the solvent background subtraction in Process C can be performed either directly with two-dimensional data from Process B or using a stored solvent scattering signal from the absolute backgrounds library. In the latter case, the data may have been stored in azimuthally averaged form (one dimensional), in which case they will need to be mapped (interpolated) to two dimensions to match the dataset dimensionality and angular range of the data processed in Process C.

Many of the corrections are multiplications and therefore follow the law of commutation. The corrections that can be commutated have been grouped together (*cf.* Fig. 1 ▸), where such a commutation would not affect the result. The commutability becomes clear when we write Process B as a pseudo-equation, with a 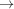 indicating a more involved operation, a 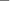 indicating a subtraction, and a 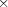 indicating a multiplication operation with either a scalar or a vector: 
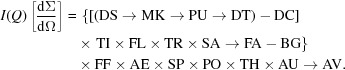



### 3.1. Regarding uncertainties   

In the modular approach presented here, it is pragmatically assumed that the correction steps and their uncertainty propagation are uncorrelated to, and independent of, any preceding or subsequent correction steps. This assumption makes the uncertainty propagation easy to implement, as each correction step can remain unaware of its position within the complete scheme. However, this assumption of uncorrelated independent uncertainties for each step is not strictly true for those correction steps that are performed in each of the processes but use the same correction factors (as is the case for FF, PO, SA and AE, for example). Their multiple occurrence within the general scheme may lead to an overestimation of the propagated uncertainty (to which the uncertainty of each of these is added several times). The propagated uncertainty estimates in the final output may, therefore, be overestimated owing to the multiple occurrences of these corrections in the schema. The magnitude of this overestimation of uncertainties can be reduced by avoiding the total number of such duplicate correction steps, by considering the ‘merging points’ in the data correction sequence.

In the data correction sequence, merging points exist at the container and solvent scattering subtraction operations, where the uncertainties of the preceding two sequences are combined into the uncertainty of the resulting data values. Operations that can be deferred beyond these merging points are performed only once on the data as opposed to twice when placed before the merging points (*i.e.* where they are applied once per dataset), and therefore the total resulting uncertainty estimate will be smaller by avoiding the duplicate operations.^3^

The corrections in this sequence have thus been ordered to limit the number of duplicate operations, while retaining the usefulness of the results. In particular the choice was made to obtain both the solvent scattering (output B) and the analyte scattering (output C) in a reusable form at the cost of retaining some duplicate operations, whereas the scattering signal from the container itself was not considered important enough to obtain in reusable form. This has the added benefit that the correction values, for *e.g.* the flatfield, do not need to be the same between processes *A* + *B* and *A* + *C*. Pragmatic choices have been made for corrections which do not benefit from an early placement in the schema but can be deferred until after a merging point, saving time and limiting the uncertainty expansion. The authors believe that the simplicity of implementation of this approximate error estimate propagation method outweighs the drawbacks of the potentially increased uncertainty estimate. The increases can also be limited by accurate determination of the correction values, so that the correction value uncertainties are small and do not contribute significantly. Finally, as multiple uncertainty estimators are provided, including one determined during the final azimuthal integration step, the user remains free to choose the final estimator (or estimator combination) that they deem to be the most accurate reflection of the practical uncertainty for their instrument. The provided propagated uncertainties can only improve with better, more involved uncertainty propagation considerations in the future.

## 4. A further practical modification   

In practice, the flux and transmission corrections can be combined. We define the transmission factor 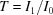, with the incident flux denoted as 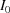 and the emergent flux (the sum of the transmitted, scattered and diffracted radiation) as 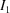. Then, defining the prior detected signal 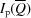 and flux- and transmission-corrected signal 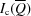, we get 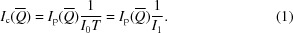
Combining these operations ostensibly negates the need for an upstream beam flux monitor, to the great relief of many instrument scientists. However, as the transmission factor still needs to be known for the self-absorption correction, their elation is likely to be short in duration.

## 5. Instrumental effects for consideration in the analysis rather than the data corrections   

There are some effects which are, unfortunately, best considered in the scattering pattern analysis procedure rather than in the data correction procedure. There are three effects: the resolution function smearing, the multiple scattering effect and the scattering length density contrast. We will discuss each of these briefly.

The resolution effect originates from the uncertainty in the scattering vector for each individual photon. Some of the origins of these scattering vector uncertainties are well defined, such as finite beam size and divergence, and the scattering vectors for an ensemble of photons will, therefore, exhibit a well defined spread. This is known as the resolution function, and it can, in principle, be corrected for. The procedure to do this can be likened to a ‘sharpening’ procedure in image processing and carries the risk of introducing artefacts due to its ill-posed nature. As it is more prominent in the neutron scattering field, a workable solution has already been developed there: the mathematically safer method for including the resolution contribution is to include the resolution function in the analysis. For this reason, the resolution function should accompany the data, for example using the provisions in the NXcanSAS format. By then convoluting or ‘smearing’ the model scattering with the resolution function, the problem is tractable and can be taken into account without reservation (Rennie *et al.*, 2013 ▸).

The same holds for the multiple scattering contribution (Warren & Mozzi, 1966 ▸). This is the probability that photons are scattered twice or multiple times, and is directly related to the scattering probability of a material for the energy used and its thickness. The multiple scattering contribution is hard to correct for in the original data. It is much easier to convolute the scattering pattern with the multiple scattering effect and likelihood, and to take it into account in that manner (Rennie *et al.*, 2013 ▸).

The last effect is the energy dependence of the scattering length density contrast. This energy dependence implies that, while the scattering vector is described independent of the energy, the scattering cross section will still be correlated, particularly near to absorption edges. There is, to our knowledge, no current solution for this, and information on the used energy must, therefore, always accompany a scattering curve.

## 6. Conclusions   

We have presented a comprehensive data correction sequence, which can be used as the core of a software implementation or as a reference correction sequence against which other, faster implementations can be proven. The sequence is chosen so that it returns useful information, in particular for dispersions, where the absolute scattering signal from the dispersant and that from the analyte are obtained independently.

By presenting this schema, we hope to encourage unity and consistency in the worldwide data correction efforts, to the betterment of the small-angle X-ray scattering field.

## Figures and Tables

**Figure 2:**
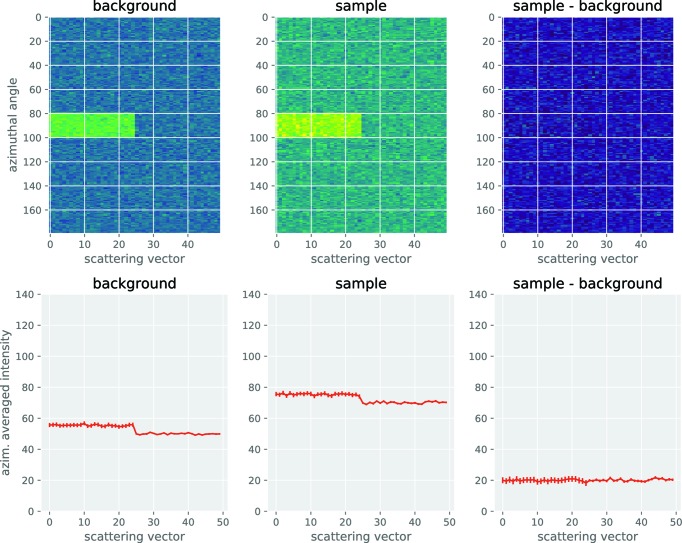
Two approaches to background subtraction. Top row: subtraction of two-dimensional images; bottom row: subtraction of the azimuthally averaged images.

**Figure 3:**
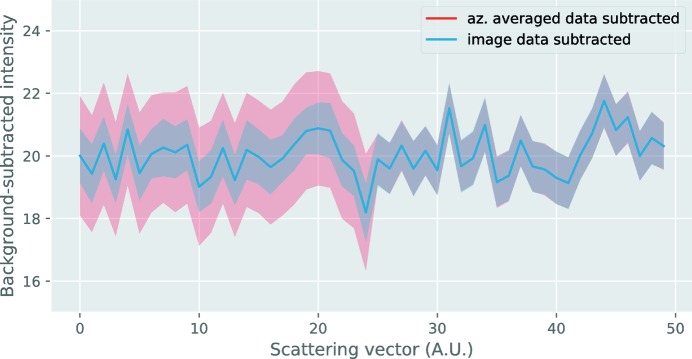
The uncertainties resulting from the two approaches, showing a lower uncertainty estimate for the image subtraction approach.

**Figure 4:**
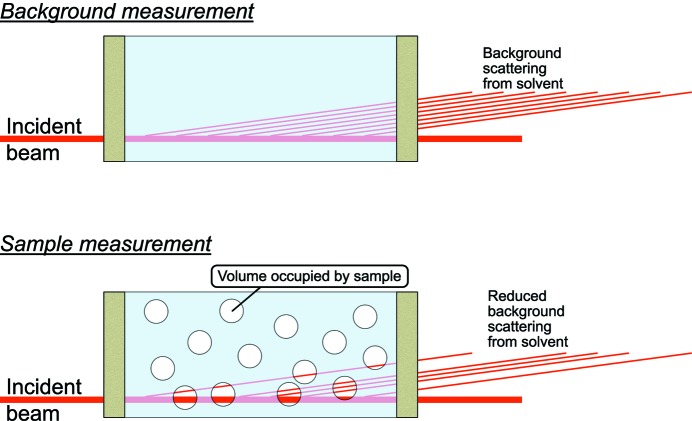
A schematic description of the displaced volume correction: a reduction in background signal when significant volume fractions of analyte are present. In that case, the thickness of background material is reduced, and its contribution to the scattering signal reduces proportionally.

**Figure 5:**
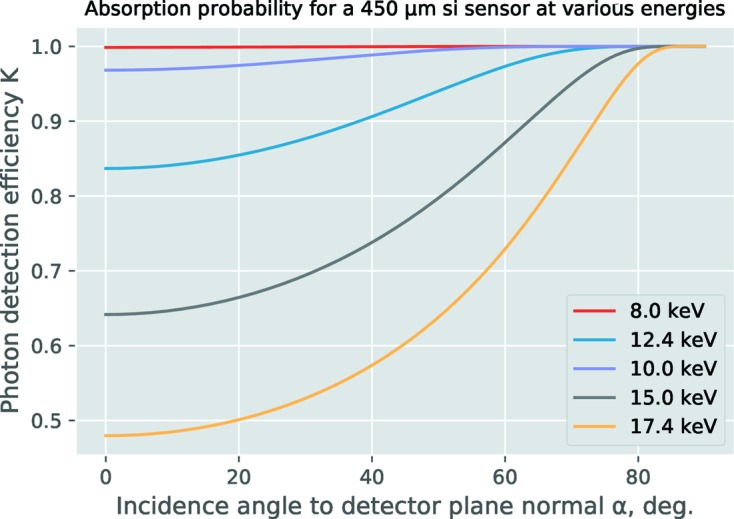
Detection efficiency of photons of various energies, dependent on their angle of incidence to the detector plane.

**Figure 6:**
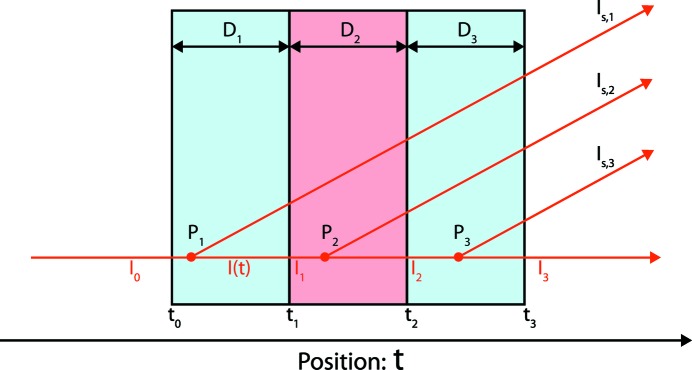
Schematic overview of the definitions used in the derivation of the background correction for dispersions ‘sandwiched’ between two container walls.

**Table 1:** Examples of measurements to be used for the various processes, for a range of sample types ‘NIB’ stands for nothing in beam. This means that there is nothing in the beam path at the sample position; the normal flight-tube exit and entrance windows, for example, are kept in place. ‘Empty cell’ can be replaced with ‘empty capillary’ if capillaries are used. For sooty flames, the non-sooty flame is a best effort, since the burning conditions, and therefore the background, are by definition different.

To determine the scattering of	Process A	Process B	Process C
Solids			
Freestanding solid (slab, plate or foil)	NIB	Solid	N/A
Metal alloy	NIB	Alloy	N/A
Nanoparticles embedded in a polymer	NIB	Polymer	Polymer with embedded nanoparticles
Porous membrane in vacuum (dry)	NIB	Membrane	N/A
Only pores in the membrane (dry)	NIB	Non-pore-containing membrane	Dried, porous membrane
Porous membrane in *in situ* cell (gas/wet)	Empty cell	Filled cell	Immersed membrane
Only pores in the membrane (gas/wet)	Empty cell	Immersed non-pore-containing membrane	Porous membrane

Powders			
Powder in between sticky tape (dry)	Sticky tape	Powder in sticky tape	N/A
Powder in *in situ* cell (gas/wet)	Empty cell	Gas- or water-filled cell	Immersed powder in cell

Liquids			
Pure liquids	Empty cell	Liquid	N/A
Nanoparticle dispersion in liquid	Empty cell	Solvent	Solvent + nanoparticles
Proteins in buffer	Empty cell	Buffer	Buffer + protein
Micelles in oil/water dispersion	Empty cell	Oil; water (separately)	Micelles in dispersion

Gases			
Pure gases	NIB or empty cell	Gas	N/A
Particles in carrier gas (flow-through cell)	Empty cell	Gas	Gas + particles
Particles in carrier gas (free flowing)	NIB	Gas	Gas + particles
Sooty flames (see note in caption)	NIB or empty cell	Non-sooty flame	Sooty flame

## Footnotes

1 The uncertainty estimates should be provided as the estimated standard deviation characterizing the width of the likelihood distribution. For the scattering cross section, this uncertainty is composed of a combination of contributions, including the propagated counting uncertainties, augmented or superseded by contributions from other local effects such as, for example, the flatfield uncertainty and background measurement uncertainty. In accordance with the NXcanSAS definition (http://download.nexusformat.org/doc/html/classes/applications/NXcanSAS.html), it is recommended to separate the local per-pixel uncertainty from the global scaling factor uncertainty.

2 The Poisson (counting) statistics denote the probability of a number of randomly arriving, discrete events occurring within a given time frame. This is used here to determine the minimum scattering signal uncertainty associated with an observed number of detected events. That can only be achieved when applied to the actually detected events, and not on an estimated number of incident photons theoretically corresponding to that number of detected events.

3 Consider, for example, the difference in this sequence with three mathematical operations, , *versus* the same with only two: .

## Appendix A Background subtraction: one *versus* two dimensions

When dealing with isotropically scattering samples, many data corrections can be performed on an image level (two dimensional) or on azimuthally averaged curves (one dimensional). In other words, the background subtraction is done either

(1) by subtracting the background image (two dimensional) from a sample measurement, followed by azimuthal averaging to obtain a one-dimensional curve, or

(2) by subtracting the azimuthally averaged background data (one dimensional) from the azimuthally averaged sample measurement,

with the latter being somewhat less computationally intensive. We can demonstrate, however, that the background subtraction in particular, and therefore all preceding operations, are best done in two dimensions in order to reduce the resulting scattering signal uncertainties. As an added big benefit, support for anisotropically scattering samples is then automatically included.

The improvement for the background subtraction procedure is due to the way the scattering signal uncertainties are calculated during the azimuthal averaging and then propagated. As uncertainty estimates play an important role in the analysis of small-angle scattering images, having access to (multiple) reasonable estimates is important. Besides the propagated Poisson uncertainties, it is possible to get a second estimate from the azimuthal averaging procedure, which will include more instrumental contributions than Poisson uncertainties alone. This second estimate is obtained by calculating not only the mean scattering signal but also the standard error on the mean.

To demonstrate how the choice of dimensionality affects the resulting scattering signal uncertainties, we simulate both a background image and a sample measurement image. For simplicity, these are simulated in polar format: *Q* varies along one axis and the azimuthal angle along the other. A systematic instrumental contribution is added to the image, representing, for example, a small contribution from scattering off the edge of the collimation system or beamstop. Similar scattering can originate from capillary walls or imperfections in window material.

We create two images, 180 by 50 pixels in size, that represent the background and sample measurement in azimuthal angle *versus* scattering vector. The background image contains 50 counts with Poisson noise and a central region of pixels with 50 more noisy counts. The sample image is set up in a similar way, with 70 noisy counts (20 signal counts on top of the 50 background counts), and the central region of scattering signal elevated by another 50 counts as in the background.

Approach 1 is the calculation of the azimuthal average and the standard error on the mean from the sample and background images directly, followed by subtraction and propagation of the uncertainties (top row of Fig. 2 ▸).

Approach 2 involves subtraction of the background image from the sample image, followed by the calculation of the azimuthal average and the standard error on the mean (bottom row of Fig. 2 ▸).

In conclusion (Fig. 3 ▸), we see that both approaches result in the identical retrieval of 20 mean counts of signal after subtraction of the background. However, the region of elevated signal results in a much higher uncertainty estimate on the scattering signal which has been background subtracted after azimuthal averaging. If we do image subtraction of the background before averaging, such spurious signals can – if they are stable in both measurements – leave no large detrimental effect.

## Appendix B The displaced volume correction: DV

The displaced volume correction is one that only needs to be considered for measurements on sample dispersions with a high volume fraction of analyte in the matrix. A rule of thumb would be to use this for analyte volume fractions of at least 1%.

In these cases there is a reduction in the amount of background material that the primary beam passes through, since a part of that space is now no longer occupied by the background material (see Fig. 4 ▸). In other words, there is a reduced length of background material in the beam, leading to a reduction in the background signal by an amount proportional to the volume fraction of sample in the beam. Perhaps counter-intuitively, this is not something that is compensated for by the transmission measurement; the sample may have an identical overall absorption probability to the background but still occupy a large fraction of the space.

What complicates matters is that this only reduces the background signal originating from the solvent, while leaving the background signal from the sample container walls unaffected. This means that the background signal needs to be disassembled into its components and that the background scattering signal from the liquid needs to be reduced in a scaling procedure. For this reason, the schema in Fig. 1 ▸ has two background subtractions, the first to separate the solvent as well as the dispersion signal from the capillary walls, and the second to subtract the solvent signal from the solvent + analyte signal. Before applying the second subtraction, the solvent signal is multiplied with its (remaining) volume fraction. In this case, the scattering signal of the sample alone is obtained.

The second complication is that there is something of a chicken-and-egg problem; you cannot do this correction without knowledge of the volume fraction occupied by the sample. That volume fraction, however, may result from the scattering pattern analysis of the corrected scattering pattern (which you do not have yet). It may be possible to apply this correction in an iterative manner (an approach as yet untested). Alternatively, the volume fraction of analyte needs to be determined using other methods.

The correction of the observed solvent scattering signal  to the corrected solvent scattering signal  using the volume fraction of analyte  then becomes

This corrected signal can be subtracted from the signal of the dispersed analyte in the solvent.

This correction will be significant if (1) the analyte volume fraction is significant, *i.e.* larger than 1 vol.%, and (2) the scattering signal from the sample is weak compared to the signal from the solvent. Proteins in solution and micellar systems are a prime example, but also dispersed polymers and vesicles may be affected.

## Appendix C The angle-dependent efficiency correction: AE

One additional correction can be considered, which takes into account the variation in detection probability of a photon passing through the detection layer at various angles (Zaleski *et al.*, 1998 ▸). When a photon passes through the detector at an angle perpendicular to the sensor surface, its detection probability is proportional to the absorption probability. This is, then, a function of the linear absorption coefficient (and thus the photon energy and sensor material) and the thickness of the sensor layer. If the photon passes through the detector layer at an angle, the amount of material it passes through is greater and the detection probability increases. This means that the detection efficiency of a photon is greater when it arrives at oblique angles rather than perpendicular to the surface.

We ignore here, for simplicity, the fact that the detector surface is divided into individual voxels, each of which can absorb all or some of the photons that are passing through. The probability of absorption within such a voxel is also dependent on its exact shape, the incidence vector and the energy of the photon. The probability of detection is furthermore dependent on the localized probability for charge sharing between neighbouring pixels and the energy threshold settings used for that pixel (Kraft *et al.*, 2009 ▸; Bergamaschi *et al.*, 2015 ▸). Lastly, at lower energies, photons arriving at oblique angles are more likely to be absorbed by the deadlayer of the sensor, reducing their probability of detection compared to photons arriving perpendicular to the detector surface (Wernecke *et al.*, 2014 ▸). These additional complications could be considered in future fine-tunings of the AE correction.

This angle-dependent efficiency correction could be considered part of the flatfield response correction of the detector. Its source, however, is not detector imperfections but lies in the instrument geometry coupled with the detector sensor thickness. This correction can, therefore, be considered separately. Since its magnitude can be easily estimated, it is straightforward to take it into account. If we rewrite the derivation from Zaleski *et al.* (1998 ▸) to let *K* represent the mass energy-absorption efficiency of a detector surface of thickness *d* as a function of the angle of incidence α of a photon to the detector surface normal, we get

where  is the mass energy-absorption coefficient for silicon for a given energy. The correction of the observed scattering signal  to the corrected scattering signal  then becomes

The magnitude of this correction becomes larger with increasing energy, thinner detector surfaces and increased angular coverage of the detectors. Fig. 5 ▸ shows the magnitude for various energies for a typical sensor thickness of 450 µm. Its magnitude may not be large for SAXS experiments, but it is easy to implement and correct for. Furthermore, when combining SAXS with wide-angle X-ray scattering data, the correction becomes more important.

## Appendix D Deriving the background subtraction sequence

Dispersions are often measured inside a sample container (indeed, it is hard to do otherwise). This implies that we have an absorbing container wall upstream and downstream, between which we have a particular length of sample, which also absorbs. Here, we show how to extract the sample scattering in this geometry. This derivation forms the basis of the data correction sequence in the manuscript. For this calculation, the self-absorption correction of the scattered radiation is not considered.

### d1. Base definitions

Note that the definitions made herein are for this appendix only and do not apply to the general manuscript.

#### d1.1. System definitions

The scattering system is considered to consist of a three-component sandwich-like structure: an upstream sample container wall, followed by a sample, followed by a downstream sample container wall (see Fig. 6 ▸). All components are considered to be plate like in shape, with the plate normal parallel to the direct beam. Furthermore, the distance between the sample and the detector is considered to be much larger than the thickness of the sample.

#### d1.2. Geometric definitions

The upstream sample container wall is denoted by the subscript 1, the sample by subscript 2 and the downstream sample container wall by subscript 3. The following definitions are made:

*D*: the thickness of a phase.

*t*: the running variable of distance travelled through all phases.

: position at the start of the upstream sample container component.

: position at the start of the sample component (end of the upstream sample container component).

: position at the start of the downstream sample container component (end of sample component).

: position at the end of the downstream sample container component.

: the scattering probability of phase *n*.

: the primary beam flux at position *t*.

: the scattered flux at position *t*.

: the primary beam flux.

: the primary beam flux entering the sample phase.

: the primary beam flux entering the downstream sample container component.

: the primary beam flux after absorption through all of the components.

: the linear absorption coefficient of phase *n*.

: the angle of the scattered radiation.

: the transmission factor of a given phase or set of phases.

### d2. The derivation

#### d2.1. Absorption of the unscattered beam

The X-ray absorption is defined as

The beam intensities entering and exiting the various phases therefore work out as

#### d2.2. Absorption of the scattered beam by subsequent components

In this geometry, the probability of absorption of a scattered photon increases with the scattering angle, as it has to travel through more material. The length of travel of the photon though subsequent materials is defined as

The transmission factor *T* of scattered radiation through subsequent phases therefore is

#### d2.3. Flux of the scattered beam in the scattering component

The scattered flux and direction-dependent transmission factor have been derived elsewhere (Pauw, 2013 ▸) and found to be

For the initial derivation, however, we do not consider the scattering-angle-dependent increase in material path length, so the term .

#### d2.4. Flux scattered from component phases

The scattered fluxes of the individual components are defined as follows:

With , this simplifies to

#### d2.5. Flux scattered from the total

The total scattered flux is the sum of the scattering from all three components in the beam, attenuated by their subsequent phases:

Replacing the components of equation (12) with equations (11), (6) and (8), we get for the total scattered flux of both sandwich-cell walls and the intermediate sample

Assuming phases 1 and 3 are identical, this simplifies to

#### d2.6. Determining *P*_1_

Before we can continue, we must find out how to determine . We do this in a background measurement, by measuring the scattering from the empty cell  (in practice, the cell is ideally drawn to a vacuum, although the signal from air is assumed to be negligible). This implies that  and  are both zero as this phase is not present in the measurement. We then obtain  from equation (13):

(note that the first factor 2 originates from considering the upstream and downstream walls separately), so

#### d2.7. Extracting *P*_2_

Finally, we want to find the scattering probability of phase 2,  (which is what we are really seeking), by rearranging equation (14):

Substituting the transmission factors for the empty cell  and cell plus sample  , we arrive at the (more or less) standard background subtraction calculation:

So, even when we thoroughly consider the scattering process of a sample sandwiched between two sample cell walls, we arrive at a simple equation for determining the sample scattering probability from the total measured scattering signal.

### d3. Final remarks

There are interesting aspects when we use this background subtraction equation in practice. Firstly, we find that it is not necessary to determine the sample cell wall thickness . Secondly, both the sample measurement and the background measurement are normalized to the thickness of the sample phase  only. Lastly, note that this is, of course, only valid if the same sample cell is used for both the background and the sample measurement.

Equation (18) as derived thus is represented using the modular data corrections as shown in Fig. 1 ▸. The thickness correction occurs after background subtraction, and the transmission and incident flux corrections have been applied before subtraction. The same background equation also works for simpler cases, for example when measuring a solid sample with an empty background.
